# Neuropsychiatric Symptoms in Clinically Defined Parkinson's Disease: An Updated Review of Literature

**DOI:** 10.1155/2022/1213393

**Published:** 2022-05-09

**Authors:** Paloma Macías-García, Raúl Rashid-López, Álvaro J. Cruz-Gómez, Elena Lozano-Soto, Florencia Sanmartino, Raúl Espinosa-Rosso, Javier J. González-Rosa

**Affiliations:** ^1^Institute of Biomedical Research Cadiz (INIBICA), Cadiz, Spain; ^2^Department of Psychology, University of Cadiz, Cadiz, Spain; ^3^Neurology Department, Puerta del Mar University Hospital, Cadiz, Spain; ^4^Neurology Department, Jerez de la Frontera University Hospital, Jerez de la Frontera, Spain

## Abstract

**Background:**

Neuropsychiatric symptoms (NPS) are a common and potentially serious manifestation of Parkinson's disease (PD) but are frequently overlooked in favor of a focus on motor symptomatology. Here, we conducted a literature review of the prevalence and type of NPS experienced by PD patients with a clinically defined course of their illness.

**Methods:**

We identified reports of NPS in patients with PD and mean disease duration over 3 years. Three databases—PubMed, Scopus, and Dialnet—were searched for relevant literature published between 2010 and 2020. Predefined exclusion criteria were applied prior to a descriptive analysis of the literature base.

**Results:**

In all, 87 unique reports were identified and 30 met inclusion and exclusion criteria. These included 7142 patients with PD (male: 67.3%; mean age: 66.2 years; mean disease duration: 6.7 years). The most frequent NPS were mood disorders (apathy, depression, and anxiety), psychosis, and impulse control disorders (ICD). Treatment with dopamine agonists was identified as an important risk factor for ICD. Co-occurrence of NPS and cognitive dysfunction was also evidenced in a number of studies. Patients with more significant cognitive deficits and higher levels of NPS appeared to be of older age with a longer disease duration and to have more severe motor symptoms.

**Conclusions:**

NPS, most commonly mood disorders (apathy, depression, and anxiety), psychosis, and ICDs are frequent manifestations of PD. The results of this review reflect the need to develop unified validated assessment protocols for NPS in PD, as well as to improve their management in clinical practice.

## 1. Introduction

Parkinson's disease (PD) is a neurological process of chronic course, characterized by a complex clinical pattern of motor and non-motor symptoms. The precise etiology of PD remains unknown but is thought to involve a combination of both environmental and genetic factors. PD is the second most frequent neurodegenerative disorder after Alzheimer's disease [1] with an estimated prevalence of 0.3–1.0% for the general population and an incidence of around 3.0% among individuals aged >80 years [2, 3]. PD is more prevalent among males compared with females with an incidence ratio of around 2 : 1 [3].

The main symptoms associated with PD include rigidity, bradykinesia, tremor, and instability, as a result of an impairment in the striatal dopaminergic pathway (Table 1). Dopamine-replacement therapy is the current mainstay of treatment for such symptoms. However, patients with PD also experience non-motor symptoms including cognitive and psychiatric disorders, pain, and autonomic nervous system dysfunction. The prodromal phase of the disease may extend up to 20 years before the manifestation of motor symptoms and is additionally characterized by the presence of anosmia, depression, constipation, and rapid eye movement (REM) sleep behavior disorders [4]. Although the burden of non-motor symptoms is usually larger and more prolonged than that conferred by motor symptoms, they are typically underrecognized despite their significant contribution to the functional impairment patient's experience [5].

Due to the marked functional impairment associated with PD, management strategies have focused principally on the palliation of the motor symptoms of the disease. As the disease progresses, up to 90% of patients experience some form of NPS including mood disorders, fatigue, psychosis, cognitive impairment, sleep problems, and addictions [6]. However, despite the high prevalence of the NPS and the insidious impairment provoked on patients' and their caregivers' quality of life (QoL), there is no standardized evaluation criteria for NPS in clinically defined PD. Importantly, the manifestation of NPS during the different stages of the disease and the impact of current treatments for motor symptoms on NPS in these patients is not well defined. For this reason, we undertook a review of the recent literature on the prevalence and nature of NPS during the initial years following a diagnosis of PD in order to inform rational consideration of appropriate treatments and management strategies to address all the manifestations of this progressive and debilitating disease.

The objective of the present literature review and descriptive analysis was to summarize the prevalence, nature, and the current stage of NPS among patients with PD, specifically focusing on recent studies involving patients experience during the initial years following their clinical diagnosis. Additional objectives were to identify the most common NPS among PD patients in relation to their clinical characteristics and the prevalence of cognitive impairment and to explore the relationship between NPS and current approaches to the treatment of PD.

## 2. Methods

### 2.1. Identification of Relevant Literature

The literature was systematically searched on January 13–15, 2020, and again on December 18, 2020, taking into account only articles published in peer-reviewed journals. Three electronic databases—PubMed, Scopus, and Dialnet (Table 2)—were chosen according to the following factors: accessibility, availability, and relevance for the research question to be addressed. The Boolean operators used to search the databases are detailed in Table 3. Duplicate reports were removed and predefined inclusion criteria applied to identify relevant reports as follows:

- Clinical studies of patients with a diagnosis of PD and with a mean *disease duration* over *3* years. This criterion was applied in order to reduce a potential confounding effect due to the subsequent development of other neurological signs and to ensure the exclusion of patients with atypical PD
- Published between 2010 to 2020
- Language: English and Spanish
- Participant sample of least of 30

Predefined exclusion criteria were then applied:

1. Reviews or nonexperimental articles
2. Animal studies
3. Reports not directly related to the research objectives
4. Reports not available through the specified databases
5. Participant sample<30
6. Reports of patients with PD illness duration after clinical diagnosis <3 years
7. Reports of patients diagnosed with any other disorder that could interfere in the final results

### 2.2. Analyses

A descriptive, narrative analysis of the literature base was undertaken. No meta-analyses or formal hypothesis testing was undertaken. Descriptive statistics are presented for the frequency of NPS across the reports included. Average age and disease duration were calculated using the arithmetic mean where such data were available or where it was possible to calculate them based on the data presented.

## 3. Results

**Figure 1:**
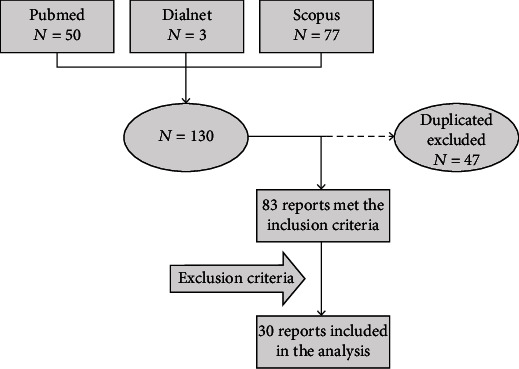
Flowchart of the search strategy and screening process.

A total of 130 articles were identified (PubMed, *n* = 50; Scopus, *n* = 77; Dialnet, *n* = 3), of which 83 were unique reports and 30 met the predefined inclusion and exclusion criteria (Figure 1). Supplementary Table 1 details the reports that were excluded from the analysis and the reasons for exclusion. The incidence of each exclusion criteria was as follows 28.1%, 5.3%, 11.5%, 1.8%, 14.0%, 38.6%, and 7.0% for exclusion criteria from 1 to 7, respectively.

The 30 reports accepted for further review included a total of 7142 patients with PD located in countries in Africa (2), Asia (5), Europe (15), North America (5), South America (1), and Oceania (2). The top-line demographics of the patient cohort for each included report are summarized in Table 4. The mean PD patient age was 66.2 ± 8.8 years, 67.3% were male, and the average duration since diagnosis of PD was 6.7 ± 4.5 years [7–36].

A summary of the neuropsychiatric assessments undertaken and the results of the assessments in each report are included in Table 5.

### 3.1. Prevalence of NPS

**Figure 2:**
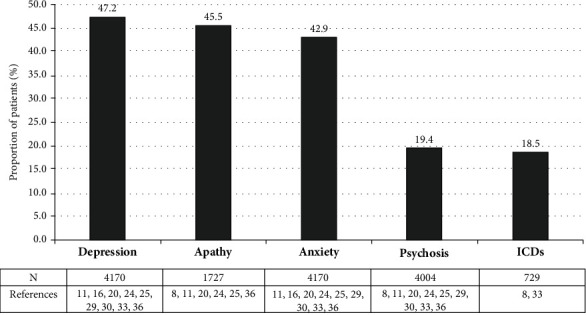
Most prevalent NPS in the PD sample. Only data from articles which reported NPS prevalence in percentage format was included in this figure [8, 11, 16, 20, 24, 25, 29, 30, 33, 36]. It should be noticed that the data reported may not have a unique origin as different instruments of assessment were used. Data reported in scoring format was not included.

The most commonly reported NPS encountered in the studies included in the current review were mood disorders, particularly apathy, depression and anxiety, psychosis, and impulse control disorders (ICDs; Figure 2).

### 3.2. Mood Disorders: Apathy, Depression, and Anxiety

The most commonly encountered mood disorders were depression (47.2%) [11, 16, 20, 24, 25, 29, 30, 33, 36], apathy (45.5%) [8, 11, 20, 24, 25, 36], and anxiety (42.9%) [11, 16, 20, 24, 25, 29, 30, 33, 36].

In one study, a cohort of 492 patients with PD found that both the presence and severity of apathy had a significant negative impact on patient QoL (as measured using the PDQ-8) [8]. The presence of apathy and mood alterations were associated with the highest correlation coefficient (0.63; *p* ≤ 0.001) and effect size (0.62; *p* ≤ 0.001) for all the NPS identified in this study (including psychotic symptoms and ICD) [8].

The relationship between depression (as measured using the UPDR-S) and the severity of PD (as measured using the Hoehn-Yahr scale) was examined in one study [30]. The presence of depression was associated with female sex (odds ratio [OR] = 1.85; *p* ≤ 0.05) and Hoehn-Yahr Scale score (Hoehn-Yahr stage III, OR =2.01, *p* ≤ 0.05; Hoehn-Yahr stages IV + V OR = 1.92, *p* ≤ 0.05) [30]. A separate investigation identified a positive relation between UPDR-S motor score and BDI score (OR = 2.9; *p* = 0.004), as well as between an increasing severity of depression and Hoehn-Yahr scale score (OR = 1.75; *p* = 0.003) [29]. However, a further study found no statistically significant correlation between depression as measured using the BDI and Hoehn-Yahr scale score (*r* = 0.01; *p* = 0.91) [22]. In an examination of depression among patients with PD with and without motor symptoms, patients with motor symptomatology were of older age compared to those without motor symptomatology (72.5 ± 9.6 vs 69.3 ± 9.2, respectively; *p* ≤ 0.01), had a longer disease duration (10.7 ± 6.9 vs 6.4 ± 5.5, respectively; *p* ≤ 0.001), and had higher PD severity (Hoehn-Yahr stage score: 2.8 ± 0.8 vs 2.3 ± 0.9, respectively; UPDRS motor score: 40.4 ± 14.5 vs 28.0 ± 13.9, respectively; *p* ≤ 0.001) [33]. Moreover, compared to patients without motor symptoms, those with motor symptoms were more likely to have depression (35.1% vs18.4%; *p* ≤ 0.01), anxiety (29.4% vs 12.6%; *p* ≤ 0.01), psychosis (hallucinations: 17.2% vs 5.7%; delusions: 13.7% vs 3.4%; *p* ≤ 0.01 for both), and dementia (19.5% vs 4.6%; *p* ≤ 0.001) [33].

In relation to Guo et al. (2015) and Solla et al. (2011), they observed that depression (53.2% and 30.9%, respectively) and anxiety (44.8% and 25.2%, respectively) were the most prevalent neuropsychiatric condition in their sample and more frequent among females [11, 33].

A number of studies examined the relationship between depression, anxiety, and apathy and a number of other factors, manifestations, and treatments including genetics [9, 12, 23, 34, 36], parkinsonian impairments [31], pharmacological treatments [35], non-pharmacological treatments [7, 13], hypertension [20] correlation with ICD [27], olfactory dysfunction [18, 32], and the comparison and comorbidity in contrast to healthy controls or baseline data [10, 17, 19, 26, 29]. Moreover, some investigations focused on the caregivers' distress as a consequence of PD [16, 25].

### 3.3. Psychosis

Psychosis is characterized by the presence of positive symptomatology (hallucinations, illusions, and delusions) and negative symptoms (mood impairment). The mean prevalence of psychosis was 19.4% [8, 11, 20, 24, 25, 29, 30, 33, 36].

Two studies reported on the rates of hallucinations and delusions among patients with PD [29, 30]. Both studies reported higher rates for hallucinations (23.8% and 11.5%, respectively) than delusions (13.5% and 2.2%, respectively). The most common types of hallucinations were visual (20.6%), somatic (13.5%), auditory (7.2%), and olfactory (1.6%) [29]. Only two studies reported the manifestation of minor hallucinations and concretely visual misperceptions [15, 30]. No information related was found in any other article included in this review.

One study reported on the rates of negative symptomatology among patients with PD and found that negative symptoms (as measures using the SANS) were more severe among those with PD than among a control group of patients without PD (SANS score for patients with PD, 23.84 ± 15.42; SANS score for controls: 2.58 ± 3.13; *p* ≤ 0.001) [22]. However, there were no significant differences between patients with PD and the control group with regard to the severity of positive symptoms (SAPS score for patients with PD, 1.36 ± 4.16; SAPS score for controls, 0.15 ± 0.43; *p* = 0.07) [22]. Moreover, no statistically significant association was identified between the presence of positive and negative symptomatology in relation to PD severity measured according to Hoehn-Yahr scale (SAPS: Hoehn-Yahr scale *r* = 0.15; *p* = 0.31; SANS: Hoehn-Yahr scale *r* = 0.12; *p* = 0.40) [22].

In a separate study, compared with PD patients without psychosis, those PD patients with psychosis were significantly older (PD + psychosis, 63.6 ± 8.0 years; PD without psychosis: 56.1 ± 11.1 years; *p* ≤ 0.05) and had a longer disease duration (PD + psychosis, 8.6 ± 3.4 years, PD without psychosis, 6.9 ± 3.5; *p* ≤ 0.05) [29]. In this cohort, no association between the manifestation of psychotic symptoms and type, dose, or combination regimen of anti-Parkinsonian drugs was identified [29]. However, there was a statistical trend toward higher daily dose of levodopa (494.3 ± 218.2 mg vs 415.3 ± 179.5 mg; *p* = 0.08) and higher levodopa equivalent daily dose (732.5 ± 508.5 mg vs 650.6 ± 423 mg; *p* = 0.38) among those with psychotic symptoms [29].

Several studies examined the influence of psychosis in their sample in relation to other factors, manifestations, and treatments including genetics [9, 12, 23, 36], non-pharmacological treatments [7], hypertension [20], correlation with motor symptoms [33], ICD [27], olfactory dysfunctions [18, 32], neurophysiology and neuroanatomy [15], and the comparison and comorbidity in contrast to healthy control subjects [19, 26, 29]. Moreover, some investigations focused on patients' QoL and caregivers' distress due to PD [8, 16, 25].

### 3.4. Impulse Control Disorders (ICDs)

The prevalence of ICDs among patients with PD was reported in two studies [8, 33]. A prevalence of 18.5% for ICDs among patients with PD was reported [8, 33]. The presence of ICDs were associated with a detrimental impact on QoL (ICD severity: PDQ8 *r* = 0.17; *p* ≤ 0.001) [8].

In a study of pathological gambling and other variants of ICD (ICD-not otherwise specified [NOS]) among patients with PD, it was observed that both conditions were associated with a longer duration of PD in comparison to PD patients without ICD (PD + pathological gambling vs PD without ICD: *p* = 0.003; PD + ICD-NOS vs PD without ICD: *p* = 0.007; PD with pathological gambling vs PD with ICD-NOS: *p* = 0.4849) [27].

The presence of ICDs was positively associated with the consumption of dopaminergic agonists (*p* = 0.003) and more severe psychotic symptomatology (PPRS: PD+ pathologic gambling vs PD without ICD: *p* = 0.004; PD + ICD-NOS vs PD without ICD: *p* ≤ 0.001) [27]. The most notable positive symptoms were visual hallucinations (PD + ICD-NOS vs PD without ICD; *p* = 0.017), paranoid ideations (PD + ICD-NOS vs PD without ICD; *p* = 0.002), and illusions (PD + ICD-NOS vs PD without ICD; *p* = 0.018) [27]. ICDs in PD are suggested to arise as a result of dopaminergic involvement in the reward circuitry. However, one study found no correlation between ICDs and dopaminergic agonists [10]. This may be explained by the short duration of this longitudinal study of 2 years.

Finally, in a study led by Solla et al. (2011), it was found that PD patients with dyskinesias (22.2%; *p* ≤ 0.001) manifested a higher frequency of ICDs than PD patients without motor complications (3.4%), PD patients with motor complications (12.2%), and PD patients with motor fluctuations (11.8%; *p* ≤ 0.001) and that 92.9% of male patients with PD showed significant manifestations of hypersexuality in contrast to females (*p* ≤ 0.01) [33].

### 3.5. Pharmacological and Non-pharmacological Treatment: Drugs and Surgeries

A number of studies examined the association between anti-Parkinson drugs and NPS. No correlation was found neither between UPDRS subscale I psychotic symptoms and dopaminergic agonists (*p* = 0.335) nor between NPS and amantadine (*p* = 0.086), monoamine oxidase B inhibitors (MAOI-B) (*p* = 0.477) or catechol-O-methyl-transferase inhibitors (COMTI) (*p* = 0.267) [17]. Similarly, Pérez-Pérez et al. (2015) undertook a head-to-head comparison of the neuropsychiatric effect of dopamine agonists—pramipexole, ropinirole and levodopa—in PD [24]. Only pramipexole was shown to exert a positive effect on NPS with a significantly lower frequency of clinically meaningful apathy (NPI apathy score ≥4) among those treated with this agent compared to those patients treated with either ropinirole or L-dopa (*p* = 0.002) [24].

Subthalamic nucleus deep brain stimulation (STN-DBS) has been shown to be an effective alternative for motor symptoms in PD, but data are lacking with regard to the impact on neuropsychiatric and behavioral complications [7]. In a study with 6 years follow-up, a significant reduction of both neuropsychiatric non-motor fluctuations in OFF (dysphoria state) (39.1% vs 10.1%, for baseline and endpoint; *p* ≤ 0.01) and ON (euphoria state) (37.7% vs 1.4, for baseline and endpoint; *p* ≤ 0.01) was noted for patients with PD following STN-DBS [7]. The frequency of apathy (2.9% vs 24.6% for baseline and endpoint; *p* ≤ 0.01), depression (5.8% vs 13%, for baseline and endpoint; *p* = 0.167), and psychosis (0% vs 5.8%, for baseline and endpoint; *p* = 0.066) increased [7]. On the other hand, hyperdopaminergic behaviors were markedly reduced in the follow-up: nocturnal hyperactivity, creativity, hobbyism, risk-taking behaviors, compulsive shopping, pathological gambling, dopaminergic addiction, and excess in motivation were significantly less common after STN-DBS surgery (*p* ≤ 0.05) [7].

Weintraub et al. (2010), Lamberti et al. (2016), and Radziunas et al. (2020) also reported on the effect of drugs and surgery on the neuropsychiatric profile of PD patients [13, 28, 35].

### 3.6. Cognition and NPS

As illustrated in Table 5, cognition was a factor which received a different approach between studies. For that reason, studies were classified according to the following criteria: “Patients received no cognitive nor neuropsychological assessment apart from the neuropsychiatric evaluation,” “Patients received a simple cognitive evaluation with MMSE and/or MoCA or a similar screening instrument apart from the neuropsychiatric evaluation. Neuropsychological assessment was not done,” and “Patients received both a cognitive evaluation with MMSE and/or MoCA or a similar screening instrument and a neuropsychological assessment apart from the neuropsychiatric evaluation.”

Petrovic et al. (2016) suggested that PD patients with clinically significant NPS were of older age (*p* = 0.02) and had a longer disease duration (*p* = 0.011), more severe PD (*p* ≤ 0.001), and worse cognition (*p* ≤ 0.001) than those without clinically significant NPS [25]. In a separate study, dementia was more likely to be present in patients with more advanced stages of PD than at early stages (Hoehn-Yahr scale; OR = 1.72) [30]. Patients with dementia were older than those without dementia (73.7 vs 69.1 years, OR =1.07) and had a higher age at PD onset (67.7 vs 63.3 years, OR = 1.82), although this last condition became practically insignificant (OR = 1.19) after statistical adjustment was made for age and severity [30]. Martinez-Martin et al. (2015) found that NPS were more common among patients with dementia than among those without dementia (*p* = 0.007) [16]. For patients with dementia, the most prevalent NPS was apathy, while depression was more frequent in patients without dementia (*p* = 0.007) [17]. However, Orfei et al. (2018) found no differences for severity of anxiety and depressive symptomatology within their experimental groups (Parkinson's Disease Dementia [PDD], multidomain Mild Cognitive Impairment PD [mdMCI-PD], single-domain Mild Cognitive Impairment PD [sdMCIPD], no Cognitive Impairment PD [noCI-PD]). However, increased apathy was observed (PDD =15.5 ± 9.5; mdMCI-PD =7.9 ± 0.6; sdMCIPD =7.2 ± 5.5; noCI-PD =6.8 ± 5; *p* ≤ 0.001) [21]. The PDD subgroup performed worse on all the neuropsychological domains in comparison with the other groups (PDD =20.7 ± 4.0; mdMCI-PD =27.1 ± 2.0; sdMCI-PD =28.0 ± 1.6; noCI-PD =29.2 ± 0.9; *p* ≤ 0.001) [21]. In a separate study, apathy was shown to be positively correlated with phonological fluency score (rho = −0.371, *p* = 0.008) and number of errors in the Stroop test (rho = 0.412, *p* = 0.004), while depression did not appear to exert an influence in any cognitive domain [31]. On the other hand, depression (GDS: rho = −0.41; *p* = 0.002), apathy (AS: rho = -0.32; *p* ≤ 0.01), and anxiety (STAI-S: rho = -0.37; STAI-T: rho = −0.40; *p* = 0.002) were identified as predictors of learning decline [26].

Regarding to the manifestation of psychotic symptomatology in PD, it was found that patients with dementia (hallucinations: 14.9%; visual misperceptions: 12.8%; paranoid symptoms: 3.5%; delusions: 1.3%) were more likely to present positive symptomatology in comparison to PD patients without dementia (hallucinations: 5.5%; visual misperceptions: 7.3%%; paranoid symptoms: 1.3%; delusions: 0.7%) [30].

Overall, the influence of PD on cognitive performance was approached by numerous investigations, tackling this issue from different perspectives [9, 10, 12, 14, 15, 17, 18, 20, 23, 27, 33, 35, 36].

## 4. Discussion

Our review of the literature has shown that NPS are a common feature of the symptomatology of PD. The most frequent NPS experienced by patients with PD are mood disorders (depression, apathy, and anxiety), psychosis, and ICDs [8, 11, 16, 20, 24, 25, 29, 30, 33, 36]. Patients with PD also experience a range of other NPS including mental fatigue [16], sleep disturbances [17, 24, 25], and irritability [16, 17, 24, 25]. Depression appears to be more likely to be manifested in female patients with PD [29, 30, 33] and those with more severe disease [29]. Similarly, anxiety seems to be more predominant in female PD patients [29, 33]. A number of studies highlighted the prevalence of psychosis (specifically symptoms) among patients with PD, most commonly among those with older age [17, 25, 29] and longer disease duration [25, 29]. No relation has been found for psychosis and PD severity [22], nor psychosis and any type of anti-parkinsonian drugs (dopaminergic agonists, amantadine, MAOI-B, and COMTI) in relation with item 2 from UPDRS subscale I scores [17].

ICD are also common among patients with PD and appear to be associated with longer PD duration, younger age, and sex [10, 27], as well as to the consumption of dopaminergic agonists [8]. This latter observation is consistent with the hyperdopaminergic symptomatology observed in a large proportion of patients with longer disease duration and a higher dopaminergic dose. Postsurgical patients with PD evidenced lower rates of hyperdopaminergic symptomatology and higher rates of hypodopaminergic symptomatology. This may be a consequence of the decrease in dopamine agonist treatment following surgery and the slow desensitization to its effect in these patients [7, 13].

A number of studies suggested that PD patients with cognitive impairment tended to be of older age and with longer disease duration [10, 17, 25] and were more likely to manifest a major motor disability [17, 25] and NPS [10, 25, 26] (in particular mood disorders—apathy, anxiety, and depression) [26]. Patients with PD and dementia tended to have a higher prevalence of apathy than those without dementia, while the most significant NPS among patients without dementia was depression [16].

When considering the results presented here, a number of limitations should be noted. Although we offer an integrating description of the principal and most frequent neuropsychiatric symptomatology in PD according to the last-decade scientific literature, the keywords used for the literature searches may not have captured the full spectrum of NPS. Future investigations should include a major variety of related terms in the search strategy in order to guarantee full coverage of all the NPS aspects.

Audiovisuals and other alternative data sources were not consulted. A particular challenge arose from the wide diversity of perspectives and experimental samples used when studying the neuropsychiatric aspects of PD highlighting the need for a more systematic approach to research in this area.

## 5. Conclusions

The results of this review of the literature support the need to evaluate and manage NPS in patients with PD during the first years following a PD diagnosis. Given the range of assessment tools currently employed to evaluate the various manifestations of NPS, there is a need to develop an unified and comprehensive approach to the assessment of NPS in patients diagnosed with PD with the development of validated tools suitable for use in routine clinical practice. Future research should seek to define the longitudinal evolution of NPS in patients with PD along with study designs to minimize the confounding effects of treatment and the presence of dementia when evaluating NPS in PD.

## Figures and Tables

**Table 1:** Parkinson's disease symptomatology (adapted from Kalia et al. (2015) [5] and Zesiewicz (2019) [1]).

Premotor symptoms	Constipation, anosmia, rapid eye movement sleep behavior disorder, and depression
Motor symptoms	Tremor, bradykinesia, postural instability, shuffling gait, stooped posture, dyskinesia, muscle rigidity, “freezing” episodes, and micrographia
Non-motor symptoms	Staring appearance, flat affect, excessive salivation, anosmia, depression, anxiety, psychotic symptoms, sleep disruption, fatigue, autonomic dysfunction, cognitive impairment, constipation, dysphagia, urinary incontinence, dysarthria, diminished speech volume, unexplained pain, and olfactory dysfunction

**Table 2:** Databases used for this review.

Database	Description	Languages
Scopus	Online multidisciplinary database driven by Elsevier. Scopus content coverage includes 75+ million records, 24,600+ active titles and 194,000+ books	English and Spanish
PubMed	Free resource for biomedical and life sciences literature. It is maintained by the National Center for biotechnology information (NCBI). PubMed includes 30+ million citations of biomedical literature	English and Spanish
Dialnet	Hispanic database which is mainly focused on Human and Social Sciences. Dialnet is managed by the Dialnet Foundation which belongs to L Rioja University	English and Spanish

**Table 3:** Boolean operators and citations identified.

Database	Boolean operators	Number of publications
PubMed	((Neuropsychiatric [Title]) AND (parkinson [Title])) AND ((“2010”[Date - Publication]: “3000”[Date - Publication])) Additional filters applied: Humans, English, Spanish, Middle Aged + Aged: 45+ years	50

Scopus	((TITLE (neuropsychiatric) AND TITLE (Parkinson)) AND PUBYEAR >2009 AND PUBYEAR <2020 AND (LIMIT – TO (SUBJAREA, “MEDI”), OR LIMIT – TO (SUBJAREA, “NEUR”) OR LIMIT – TO publications (SUBJAREA “PSYC”)) AND (LIMIT – TO (LANGUAGE, “English”) OR LIMIT – TO (LANGUAGE, “Spanish)) AND (LIMIT – TO (DOCTYPE, “ar”))	77

Dialnet	((Neuropsychiatric [Title]) AND (Parkinson [Title]) AND ((“2010”[Date – 2 Publication]: “3000”[Date – Publication]))	2
	((Neuropsiquiatrico [Title]) AND (parkinson [Title]) AND ((“2010”[Date – 1 Publication]: “3000”[Date – Publication]))	1

**Table 4:** Demographics of the patient cohort.

Reference	Groups	N	Mean age (years) + SD	Disease duration, years	UPDRS III	Hoehn-Yahr
Abbes et al. (2018) [7]	Baseline Follow-up	102 69	58.2 ± 6.6 64.5 ± 6.6	10.8 ± 3.0 16.6 ± 4.1	11.5 ± 8.0 20.0 ± 8.3	– –

Alvarado-Bolaños et al. (2015) [8]	No NPS	76	66.7 ± 10.4	6.2 ± 4.2	22.6 ± 11.6 (range 2–53)	30.3% (stage I); 68.4% (stages II–III); 1.3% (stages IV–V)
	With NPS	416	66.7 ± 10.1	7.1 ± 5.1	32.2 ± 18.6 (range 1–107)	11.3% (stage I); 76.4% (stages II–III); 12.3% (stages IV–V)

Belarbi et al. (2010) [9]	PD patients with LRRK2 G2019S mutation	23	65.9 ± 7.4	10.5 ± 3.2	—	—
	PD patients without LRRK2 G2019S mutation	48	67.2 ± 4.4	8.5 ± 2.0	—	—

Cuciureanu et al. (2019) [10]	PD patients	112	66.0	Mean age of onset 61 years old	—	—

Guo et al. (2015) [11]	EOPD LOPD	97 251	45.2 ± 9.5 66.1 ± 6.7	9.9 ± 3.9 8.4 ± 4.9	22.7 ± 12.4 29.1 ± 12.7	2.1 ± 0.7 2.4 ± 0.7

Hassin-Baer et al. (2011) [12]	PD patients with CRP ≤3	40	66.7 ± 12.9	6.5 ± 5.5	23.5 ± 12.8	Stage II (stages 2–3)
	PD patients with CRP >3	33	71.4 ± 9.2	6.9 ± 3.4	25.0 ± 11.8	Stage II (stages 2–2.5)

Lamberti et al. (2016) [13]	General PD patients Surgical PD patients	260 234	62.5 ± 8.5 57.7 ± 8.0	8.1 ± 5.4 10 ± 3.9	18.3 ± 10.6 14.0 ± 8.5	2.0 ± 0.6 2.0 ± 0.6

Lang et al. (2020) [14]	PD patients	74	70.8 ± 6.0	5.6 ± 3.9	18.8 ± 10.5	Stage I, II, and III

Lewis et al. (2012) [15]	PD patients Healthy controls	20 20	62.3 ± 5.5 65.1 ± 4.1	5.9 ± 3.1 –	23.3 ± 9.5 –	2.2 ± 0.2 –

Martinez-Martin et al. (2015) [16]	Dementia PD patients No dementia PD patients	488 94	Full sample: 70.8 ± 9.9	8.1 ± 5.6	– –	Full sample (*N* = 582): 22.8% (stage I); 46.0% (stage II); 17.8% (stage III); 10.6% (stage IV); 2.7% (stage V)

Merino-Lopez (2016) [17]	PD patients:					
	1^st^ evaluation 2^nd^ evaluation	92 29	71.4 ± 9.1 66.5 ± 8.4	8.5 ± 4.7 7.5 ± 5.0	30.3 ± 13.8 42.4 ± 15.5	2.5 ± 0.8 2.8 ± 0.7

Morley et al. (2011) [18]	UPSIT bottom median score UPSIT top median score	123 125	67.0 ± 9.5 63.0 ± 10.3	7.3 ± 5.2 6.0 ± 5.4	24.0 ± 12.0 20.0 ± 8.0	2.3 ± 0.7 2.1 ± 0.7

O'Callaghan et al. (2014) [19]	No NPS PD patients NPS PD patients Healthy controls	25 25 30	65.0 ± 8.1 66.9 ± 6.5 65.4 ± 6.0	5.72 ± 4.0 5.32 ± 3.1 –	23.8 ± 13.7 30.5 ± 13.7 –	2.0 ± 0.6 2.2 ± 0.5 –

Ojagbemi et al. (2013) [20]	PD patients Hypertension patients	50 50	65.6 ± 9.4 66.1 ± 9.2	3.4 ± 2.6 –	42.1 ± 17.8 –	– –

Orfei et al. (2018) [21]	Mild dementia PD patients	47	73.4 ± 6.4	9.0 ± 6.0	29.7 ± 15.1	2.3 ± 0.7
	Multidomain cognitive impairment PD patients	136	68.7 ± 8.4	5.6 ± 5.1	20.7 ± 12.1	1.9 ± 0.6
	Single domain cognitive impairment PD patients	5	65.4 ± 14.3	4.2 ± 4.6	23.6 ± 8.5	1.7 ± 0.4
	No cognitive impairment PD patients	197	62.6 ± 9.5	4.1 ± 3.4	16.0 ± 9.9	1.7 ± 0.5

Oruç et al. (2017) [22]	PD patients	46	69.6 ± 9.5	6.1 ± 4.6	—	30.4% (≤2, mild PD); 69.6% (>2, severe PD)
	Healthy controls	46	68.02 ± 10.36	—	—	—

Pavlova et al. (2014) [23]	Late onset PD e3/e4 Late onset PD e3/e3 Healthy controls	16 30 20	69.25 ± 6.6 69.74 ± 8.8 69.52 ± 7.3	5.69 ± 4.0 6.0 ± 4.1 –	43.8 ± 13.3 39.5 ± 14.7 –	– – –

Pérez-Pérez et al. (2015) [24]	PD patients on pramipexole PD patients on ropinirole PD patients on levodopa	250 150 115	68.9 ± 7.0 68.9 ± 8.0 69.0 ± 7.0	7.1 ± 4.0 8.0 ± 5.0 6.9 ± 4.0	– – –	2.4 ± 1.0 2.6 ± 1.0 2.4 ± 0.8

Petrovic et al. (2016) [25]	PD patients	360	63.5 ± 10.30	7.23 ± 5.12	50.9 ± 23.5	54.2% (mild PD, stage I–II); 36.4% (moderate PD, stage III); 9.4% (severe PD, stage IV–V)

Pirogovsky-Turk et al. (2017) [26]	PD patients	68	67.0 ± 7.3	6.1 ± 5.8	23.9 ± 11.9	1.5% (stage 0); 19.1% (stage I); 1.5% (stage 1.5); 60.3% (stage II); 4.4% (stage 2.5); 11.7% (stage III); 1.5% (stage IV)
	Healthy controls	30	69.1 ± 7.8	—	—	—

Pontieri et al. (2015) [27]	No ICD Pathological gambling Other variants of ICD	98 21 36	66.0 ± 9.0 58.0 ± 9.0 64.0 ± 8.0	5.0 ± 3.0 8.0 ± 5.0 7.0 ± 4.0	19.0 ± 11.9 21.5 ± 11.6 19.1 ± 12.7	1.8 ± 0.5 2.0 ± 0.5 1.9 ± 0.8

Radziunas et al. (2020) [28]	Baseline:					
	PD patients Healthy controls	22 18	58.0 ± 8.2 55.6 ± 8.1	– –	17.4 ± 6.1 –	– –
	Post-operative:					
	PD patients with no NPS	15	57.8 ± 9.1	10.4 ± 4.	16.0 ± 5.4 (ON state); 28.4 ± 8.2 (OFF state)	–
	PD patients with NPS	7	59.1 ± 7.4	13.5 ± 2.5	21.4 ± 6.5 (ON state); 36.6 ± 4.1 (OFF state)	—

Rai et al. (2015) [29]	Young onset PD Late onset PD	26 100	42.4 ± 5.7 61.0 ± 7.9	7.4 ± 3.8 7.3 ± 3.5	27.5 ± 13.2 30.4 ± 14.2	2.3 ± 0.7 2.5 ± 0.72

Riedel et al. (2010) [30]	Neither depression nor dementia Depression Dementia Both	875 167 229 178	69.7 ± 8.4 69.1 ± 8.7 73.7 ± 7.1 73.4 ± 7.7	5.5 ± 5.1 5.6 ± 4.9 5.8 ± 5.0 6.9 ± 5.5	– – – –	Full sample (*N* = 1449): 44.2% (stages I–II); 38.7% (stage III); 17.1% (stages IV–V)

Santangelo et al. (2018) [31]	PD patients PSP Multiple system atrophy	55 42 44	66.1 ± 9.7 71.2 ± 5.7 61.1 ± 8.3	5.2 ± 3.6 4.7 ± 2.9 5.6 ± 3.1	14.6 ± 9.5 – –	– – –

Stephenson et al. (2010) [32]	PD patients	100	61.5 ± 11.3	3.6 ± 3.8	Evaluated but not informed	44% (stage 1.5); 46% (stage 2); 1% (stage 2.5); 3% (stage 3)

Solla et al. (2011) [33]	PD no motor complications PD + motor complications PD + dyskinesias PD + motor fluctuations	87 262 99 254	69.3 ± 9.2 72.5 ± 9.6 72.3 ± 9.3 72.6 ± 9.6	6.4 ± 5.5 10.7 ± 6.0 13.6 ± 6.5 10.7 ± 6.0	28.0 ± 13.0 40.4 ± 14.5 39.2 ± 10.7 40.7 ± 14.9	2.3 ± 0.9 2.8 ± 0.8 3.2 ± 0.7 2.9 ± 0.8

Swan et al. (2016) [34]	Idiopathic PD patients GBA-associated PD	55 31	68.0 ± 11.4 65.6 ± 12.5	8.3 ± 7.3 8.6 ± 5.7	16.7 ± 8.7 20.4 ± 13.2	2.3 ± 1.1 2.2 ± 0.9

Weintraub et al. (2010) [35]	PD on atomoxetine PD on placebo	28 27	63.8 ± 9.5 64.9 ± 11.5	7.9 ± 6.6 5.7 ± 5.6	23.5 ± 12.7 21.6 ± 10.0	– –

Xing et al. (2016) [36]	PD + dementia PD no dementia Healthy controls	38 40 40	72.7 ± 8.0 67.6 ± 6.2 68.7 ± 6.5	9.8 ± 4.2 7.9 ± 4.3 –	22.9 ± 10.6 27.4 ± 10.3 –	– – –

CRP, C-reactive protein; GBA, glucosidase beta acid; ICD, impulse control disorder; NPS, neuropsychiatric symptoms; PD, Parkinson's disease; PSP, progressive supranuclear palsy; SD, standard deviation; UPSIT, University of Pennsylvania Smell Identification Test.

**Table 5:** Summary of results.

Reference	Domain and test	Main finding
Abbes et al. (2018) [7]^§^	General NPS: MINI; behavior: ASBPD; depression: BDI; anxiety: BAI; apathy: SAS	All ICDs (including eating behavior and hypersexuality) as well as dopaminergic addiction significantly decreased after six years follow-up (compulsive shopping: 5.8% vs 2.9%; pathological gambling: 5.8% vs 0.0%; dopaminergic addiction: 14.5% vs 0.0%; hypersexuality: 2.9% vs 4.3%). NPS fluctuations significantly improved (ON euphoria: 38% vs 1%; OFF dysphoria: 39% vs 10%), apart from apathy which increased (3% vs 25%) after surgery

Alvarado-Bolaños et al. (2015) [8]^†^	General NPS: SEND-PD; QoL and daily activities: PDQ-8	44.5% of the patients presented psychotic symptoms, 76.5% had alterations on mood/apathy domains, and 27% manifested an ICD

Belarbi et al. (2010) [9]^§^	General NPS: NPI; cognition and dementia: FAB; depression: HDRS and MADRS	LRRK2 G2019S carriers were more likely to have depression (65% vs 39.6%) and hallucinations (26% vs 6%) than non-carriers. LRRK2 G2019S carriers had more sleep disorders (65% vs 39.6%), probably in relation to the depressive symptomatology

Cuciureanu et al. (2019) [10]^‡^	Depression: HDRS; ICDs: QUIPRS; QoL and daily activities: GAF	The ICD gravity—specially shopping, hobbyism, and punding —positively correlated with the disease duration. Patients with higher scores on the HADRS also manifested more shopping compulsions. Hypersexual behavior seemed to be dependent on age and male gender. Depression seemed to be connected to female gender

Guo et al. (2015) [11]^‡^	General NPS: NPI; behavior: FBI; cognition and dementia: ACE-R, FAB	Neuropsychiatric symptomatology was strongly associated with frontal behavioral changes (NPI, FAB, *r* = 0.661; *p* ≤ 0.001). Negative correlations between NPI scores and worse cognition (NPI, ACE-R, *r* = −0.218; *p* ≤ 0.001) and frontal lobe function (NPI, FAB, *r* = −0.212; *p* ≤ 0.001) were also found

Hassin-Baer et al. (2011) [12]^§^	Anxiety: AS; cognition and dementia: FAB; depression: BDI; psychosis: PPRS	No significant differences were found between the two groups (CRP ≤3 and CRP >3) in depression, psychosis, dementia, cognitive decline, or frontal lobe dysfunction. Reported depression (present or past) was more frequent in patients with CRP >3 than those with CRP ≤3 (54.5% vs 25%, respectively)

Lamberti et al. (2016) [13]^‡^	Apathy: LARS and SAS; behavior: ASBPD; depression: BDI and MADRS	Dopaminergic addiction (general PD patients: 0.8% vs surgical patients: 10.7%), nocturnal hyperactivity (8.9% vs 17.1%), excessive hobbyism (7.7% vs 19.2), “excess in motivation” (4.6% vs 23.9%), and psychic OFF (17.3% vs 44.0%) and psychic ON (8.5% vs 22.7%) fluctuations were more frequent in surgical candidates. Depressed mood prevailed in the general PD population (16.9% vs 10.3%)

Lang et al. (2020) [14]^‡^	Cognition and dementia: MBI-C	Commonality analysis can demonstrate the variance in the connectome between motor, neuropsychiatric. and cognitive symptomatology characteristic of PD. The caudate nucleus was identified as the epicenter of PD's symptomatology network. Neuropsychiatric impairment was associated to the connectivity in the caudate-dorsal anterior cingulate and caudate-right dorsolateral prefrontal-right inferior parietal circuits. Caudate-medial prefrontal connectivity showed a unique effect of both neuropsychiatric and cognitive impairment

Lewis et al. (2012) [15]^§^	General NPS: SCOPA-PC; anxiety and depression: HADS	NAA/Cr ratios were registered as lower in patients with hallucinations than in those without them, within the ACC, but no differences were in the PCC. Lower NAA/Cr ratios, more severe psychotic symptomatology, and a poorer performance on the Bistable percept paradigm—a neuropsychological test for visual hallucinations—were significantly correlated

Martinez-Martin et al. (2015) [16]^‡^	General NPS: SEND-PD; cognition and dementia: MMSE	The most prevalent NPS were depression (66%), anxiety (65%), and mental fatigue (57%). NPS were more predominant in patients with dementia (16%) than in patients without dementia

Merino-Lopez (2016) [17]^§^	General NPS: NPI; depression: GDS, HDRS; cognition and dementia: MMSE	92 patients with PD were followed up for >10 years. The final evaluation only referred to 29 patients. Hallucinations were significantly present in the final phase of this investigation, and they were more likely to be associated with the cognitive impairment suffered by the patients than with the collateral effects of the antiparkinsonian drugs. Depression was significantly present since the initial phase of the investigation; otherwise, it did not manifest an increase over time. Caregivers reported higher scores on apathy, anxiety, and depression items

Morley et al. (2011) [18]^§^	Anxiety: AS, STAI; depression: GDS-15, IDS; psychosis: PPRS	No significant correlation was found between olfaction and mood measures. Nevertheless, patients with UPSIT scores below the median were more likely to manifest (visual) psychotic symptomatology (30% vs 12% of the total of each group). Worse olfaction was associated with lower scores on memory and executive performance tests

O'Callaghan et al. (2014) [19]^‡^	Behavior: CBI-R	PD patients with NPS had higher scores on the subscales of abnormal behavior, mood, stereotypic motor behavior and motivation than the two other groups (controls and PD without NPS).

Ojagbemi et al. (2013) [20]^‡^	General NPS: NPI	PD patients were compared with demographically matched hypertension patients (control group) There were significant differences in the frequency of NPS manifestations between both groups (*p* ≤ 0.05), and the presence of these symptoms is associated with caregivers' distress. Severity of motor symptoms correlated with total NPI severity scores (*p* ≤ 0.001)

Orfei et al. (2018) [21]^§^	Anxiety: HAM-A; apathy: ARS and SHaPS; depression: BDI	Diagnosis of anosognosia for non-motor symptoms was more frequent in PD patients with mild dementia (36%) or multi-domain cognitive impairment PD patients (16%)

Oruç et al. (2017) [22]^‡^	Depression: BDI; psychosis: SANS and SAPS	PD patients manifested higher rates of depression and negative symptomatology than healthy controls. Results presented no differences in different stages of PD

Pavlova et al. (2014) [23]^§^	General NPS: NPI	Patients with the e4 allele showed some significant differences in their cognitive, motor and neuropsychiatric behavior. Late onset PD patients with the e4 allele had a tendency for a higher manifestation of depression, with reports of delusions and euphoria

Pérez-Pérez et al. (2015) [24]^‡^	General NPS: NPI	Only 65.2% of the patients who were treated with pramipexole (47% out of 250 patients) showed clinically significantly lower total scores than those who received ropinirole as treatment (69.3% out of 115 patients). Patients on pramipexole manifested a significant lower frequency for apathy (11.2%) than those who were on ropinirole (20.3%) and levodopa (23.8%). No other significant differences were found in NPI subscores between groups

Petrovic et al. (2016) [25]^‡^	General NPS: NPI	89% of patients manifested at least one NPS. This manifestation was significant only for the 58% of the cases. Most common NPS: anxiety (73.1%), depression (64.7%), apathy (51.7%), and nighttime disturbances (51.3%). Least common NPS: euphoria (0.3%) and delusions (1.7%). NPS positively correlated with older age and major cognitive and motor impairment. The full sample could be categorized into three different clusters: cluster 1, with no or few NPI symptoms (55.6%); cluster 2, with mild to moderate depression, anxiety and apathy (38.9%); and cluster 3, with agitation, disinhibition and irritability (5.6%)

Pirogovsky-Turk et al. (2017) [26]^§^	Anxiety: AS and STAI; depression: GDS	Clinically significant differences were found in the frequency of depression, anxiety, and apathy between PD patients and healthy controls. Anxiety and depression at baseline behaved as the best predictors for longitudinal decline on measures of verbal and visual learning. No significant correlations were found for the healthy control group

Pontieri et al. (2015) [27]^§^	General NPS: SCID-P based on DSM-IV criteria; anxiety: HARS, apathy: ARS and SHaPS; depression: HDRS; psychosis: PPRS; QoL and daily activities: ERS	Pathological gambling patients manifested higher severity of depressive and anxious symptomatology. Pathological gambling and “other variants of ICD” subjects had more severe psychotic symptoms. No correlation was found between ICD and cognitive performance for PD patients without dementia

Radziunas et al. (2020) [28]^§^	Psychosis: 4AT	Volumetric analysis revealed significant differences in cortical thickness between the two STN-DBS postoperative groups (with and without neuropsychiatric complications) in 13 gyruses on the right hemisphere and in 7 gyruses on the left hemisphere. White matter volume analysis revealed its reduction in the left caudal middle front area. These two facts might explain the enrolment of this area in the postoperative neuropsychiatric complication risk as the most insidious. NPS in STN-DBS postoperative patients may be associated with the excitation of frontal-striatum-thalamus and temporal-parietal circuits

Rai et al. (2015) [29]^‡^	Anxiety: HARS; depression: BDI; psychosis: BSRS	64% of the total sample manifested at least one comorbidity (depression, psychosis, or anxiety). NPS prevalence in the total sample: depression (43.7%), suicidal risk (31%), psychosis (23.8%), anxiety (35.7%), visual hallucinations (20.6%), tactile hallucinations (13.5%), auditory hallucinations (7.2%), and olfactory hallucinations (1.6%). Depression was more likely to be manifested in patients with higher disability, psychosis, longer disease duration, and older age

Riedel et al. (2010) [30]^‡^	General NPS: CIDI and NPI; depression: MADRS	71% of the total of patients with PD had at least one NPS: dementia 29%; depression, 25% anxiety, 20%; and psychotic syndromes, 12.7%. Depression was related to gender and Hoehn-Yahr scale score, while dementia was associated with age. Comorbidity rates for depression and dementia were mostly determined by PD severity

Santangelo et al. (2018) [31]^§^	Apathy: AES; depression: BDI-II	Apathy and depression were more severe in progressive supranuclear palsy (57.1%; 52.9%) and multiple system atrophy (35.7%; 52.6%) groups than in PD patients (7.1%; 0%)

Solla et al. (2011) [32]^‡^	General NPS: DSM-IV criteria, clinical criteria, andMINI	Patients with motor complications manifested a higher frequency of dementia (4.6%), anxiety (12.6%), depression (18.4%), and psychosis. Patients with motor complications (12.2%) and dyskinesias (22.2%) showed a higher frequency of ICDs. Patients with dyskinesias were more likely to manifest hypersexuality (8.1%) and compulsive shopping (4%), as well as dopamine dysregulation syndrome (8.1%), hallucinations (28.3%), and delusions (except of delusional jealousy) (19.2%)

Stephenson et al. (2010) [33]^‡^	No mentioned	Severity of olfactory impairment early in the disease course may behave as a useful marker for a later risk of presenting neuropsychiatric complications in PD

Swan et al. (2016) [34]^‡^	Anxiety: STAI; depression: BDI	In univariate comparisons, GBA-PD showed higher rates of depressive symptomatology (33.3%) than idiopathic PD patients (13.2%). In regression models, age, sex, disease duration, motor disability, and MoCA scores were controlled. The odds of depression were higher for GBA-PD patients vs idiopathic PD patients (OR 3.66). GBA1 mutations were associated with a greater risk of NPS comorbidity in PD

Weintraub et al. (2010) [35]^‡^	Anxiety: AS and STAI; depression: GDS and IDS	No between-group differences were found in response rates for depression (22.7% vs 9.5%, for atomoxetine and placebo, respectively). Therefore, atomoxetine was not effective for depression in PD. Neither anxiety nor apathy rates showed variation between both groups. Nevertheless, patients on atomoxetine showed a significant improvement in global cognition and daytime sleepiness

Xing et al. (2016) [36]^§^	General NPS: NPI; cognition and dementia: CDR	PDD patients manifested significantly increased plasma ceramide levels. C14:0, C24:1, and verbal memory showed negative correlations. Hallucinations, anxiety, and sleep behavior disturbances were, respectively, associated with C22:0, C20:0, and C18:0 when confounding factors were controlled

^†^Patients received no cognitive nor neuropsychological assessment apart from the neuropsychiatric evaluation. ^‡^Patients received a simple cognitive evaluation with MMSE and/or MoCA or a similar screening instrument apart from the neuropsychiatric evaluation. Neuropsychological assessment was not done. ^§^Patients received both a cognitive evaluation with MMSE and/or MoCA or a similar screening instrument and a neuropsychological assessment apart from the neuropsychiatric evaluation. 4AT: Test for Delirium and Cognitive Impairment, ACE-R: Addenbrooke's Cognitive Examination-Revised, AES: Apathy Evaluation Scale, ARS: Apathy Rating Scale, AS: Anxiety Scale, ASBPD: Ardouin Scale of Behavior in Parkinson's Disease, BAI: Beck Anxiety Inventory, BDI – II: Beck Depression Inventory II, BDI: Beck Depression Inventory, BSRS: Brief Psychiatric Rating Scale, CBI-R: Cambridge Behavioural Inventory-Revised, CDR: Clinical Dementia Rating Scale, CIDI: Composite International Diagnostic Interview, ERS: Euro-QoL Scale, FAB: Frontal Assessment Battery, FBI: Frontal Behavior Inventory, GAF: Global Assessment of Functioning Scale, GDS-15: Geriatric Depression Scale, HADS anxiety: Hospital Anxiety and Depression Scale, HADS depression: Hospital Depression and Depression Scale, HARS: Hamilton Anxiety Rating Scale, HDRS: Hamilton Depression Rating Scale, IDS: Inventory of Depressive Symptomatology, LARS: Lille Apathy Rating Scale, MADRS: Montgomery and Asberg Depression Rating Scale, MBI-C: Mild Behavioural Impairment Checklist, MINI: Mini-International Neuropsychiatric Interview, MMSE: Mini-Mental State Examination, MoCA: Montreal Cognitive Assessment, NPI: Neuropsychiatric Inventory, PDQ-8: Parkinson's Disease Questionnaire Short Form, PPRS: Parkinson Psychosis Rating Scale, QUIPRS: Questionnaire for Impulsive-Compulsive Disorders in Parkinson's Disease Rating Scale, SANS: Scale for the Assessment of Negative Symptoms, SAPS: Scale for the Assessment of Positive Symptoms, SAS: Starkstein Apathy Scale, SCID-P: Structured Clinical Interview for DSM-IV-TR Axis I Disorders, SCOPA-PC: Scales for Outcome in PD-Psychiatric Complications, SEND-PD: Scale for Evaluation of Neuropsychiatric Disorders in Parkinson's Disease, SHaPS: Snaith-Hamilton Pleasure Scale, STAI: Spielberger State-Trait Anxiety Inventory.

## Data Availability

The data used to support the findings of this study are available from the corresponding author upon request.

## Abbreviations

AES: Apathy Evaluation Scale
ARS: Apathy Rating Scale
AS: Anxiety Scale
ASBPD: Ardouin Scale of Behaviour in Parkinson's Disease
BAI: Beck Anxiety Inventory
BDI: Beck Depression Inventory
BDI-II: Beck Depression Inventory II
BSRS: Brief Psychiatric Rating Scale
CBI-R: Cambridge Behavioural Inventory-Revised
CDR: Clinical Dementia Rating Scale
CIDI: Composite International Diagnostic Interview
COMTI: Catechol-O-methyl-transferase inhibitors
ERS: Euro-QoL Scale
ESS: Epworth Sleepiness Scale
GAF: Global Assessment of Functioning Scale
GDS-15: Geriatric Depression Scale
HADS: Hospital Anxiety and Depression Scale
HARS: Hamilton Anxiety Rating Scale
HDRS: Hamilton Depression Rating Scale
ICD: Impulse control disorders
IDS: Inventory of Depressive Symptomatology
LARS: Lille Apathy Rating Scale
MADRS: Montgomery and Asberg Depression Rating Scale
MAOI-B: Monoamine oxidase B inhibitors
MBI-C: Mild Behavioural Impairment Checklist
MMSE: Mini-Mental State Examination
MoCA: Montreal Cognitive Assessment
NOS: ICD-not otherwise specified
NPI: Neuropsychiatric Inventory
NPS: Neuropsychiatric symptoms
PD: Parkinson's disease
PDQ-8: Parkinson's Disease Questionnaire Short Form
PPRS: Parkinson Psychosis Rating Scale
QoL: Quality of life
QUIPRS: Questionnaire for Impulsive-Compulsive Disorders in Parkinson's Disease Rating Scale
REM: Rapid eye movement
SANS: Scale for the Assessment of Negative Symptoms
SAPS: Scale for the Assessment of Positive Symptoms
SAS: Starkstein Apathy Scale
SCID-P: Structured Clinical Interview for DSM-IV-TR Axis I Disorders
SCOPA: Scales for Outcome in PD-Psychiatric Complications
SEND-PD: Scale for Evaluation of Neuropsychiatric Disorders in Parkinson's Disease
SHaPS: Snaith-Hamilton Pleasure Scale
STAI: Spielberger State-Trait Anxiety Inventory
STN–DBS: Subthalamic nucleus deep brain stimulation
UPDR-S: Unified Parkinson's Disease Rating Scale
ZCIB: Zarit Caregiver Burden Inventory.

## Supplementary Materials

Supplementary Materials Supplementary Table 1: excluded articles.
