# Supplementary material for: Neuropsychiatric Symptoms in Clinically Defined Parkinson's Disease: An Updated Review of Literature
Source: Behav Neurol. 2022 May 9;2022:1213393. doi: 10.1155/2022/1213393 (PMC9110237; doi:10.1155/2022/1213393)
Supplement: Supplementary Materials — Supplementary Table 1: excluded articles. [file 1213393.f1.docx]

**Supplementary Table 1. Excluded articles**

| **Title** | **Exclusion criteria**^†^ |
| --- | --- |
| Aarons, S., Peisah, C., & Wijeratne, C. (2012). Neuropsychiatric effects of P arkinson's disease treatment. *Australasian journal on ageing*, *31*(3), 198-202. | 1 |
| Abrantes, A. M., Friedman, J. H., Brown, R. A., Strong, D. R., Desaulniers, J., Ing, E., ... & Riebe, D. (2012). Physical activity and neuropsychiatric symptoms of Parkinson disease. *Journal of Geriatric Psychiatry and Neurology*, *25*(3), 138-145. | 6 |
| Adis Medical Writers dtp@ adis. com. (2019). Regularly monitor neuropsychiatric symptoms in Parkinson’s disease and adjust treatment as necessary. *Drugs & Therapy Perspectives*, *35*, 119-123. | 1 |
| Aidi-Knani, S., Regaya, I., Amalric, M., & Mourre, C. (2015). Kv4 channel blockade reduces motor and neuropsychiatric symptoms in rodent models of Parkinson’s disease. *Behavioural pharmacology*, *26*(1 and 2-Special Issue), 91-100. | 2 |
| Anderson-Mooney, A. J., Guller, L., Combs, H. L., & Dunham, K. J. (2016). Neurocognitive & neuropsychiatric phenotypes of PARK2-associated early-onset Parkinson's disease in two siblings. *Clinical neurology and neurosurgery*, *142*, 137-139. | 1 |
| Balestrino, R., & Martinez-Martin, P. (2017). Reprint of “Neuropsychiatric symptoms, behavioural disorders, and quality of life in Parkinson's disease”. *Journal of the neurological sciences*, *374*, 3-8. | 1 |
| Bickel, S., Alvarez, L., Macias, R., Pavon, N., Leon, M., Fernandez, C., ... & Litvan, I. (2010). Cognitive and neuropsychiatric effects of subthalamotomy for Parkinson’s disease. *Parkinsonism & Related Disorders*, *16*(8), 535-539. | 5 |
| Bougea, A., Stefanis, L., Paraskevas, G. P., Emmanouilidou, E., Efthymiopoulou, E., Vekrelis, K., & Kapaki, E. (2018). Neuropsychiatric symptoms and α-Synuclein profile of patients with Parkinson’s disease dementia, dementia with Lewy bodies and Alzheimer’s disease. *Journal of neurology*, *265*(10), 2295-2301. | 5,7 |
| Chiu, P. Y., Tsai, C. T., Chen, P. K., Chen, W. J., & Lai, T. J. (2016). Neuropsychiatric symptoms in Parkinson’s disease dementia are more similar to Alzheimer’s disease than dementia with Lewy bodies: a case-control study. *PloS one*, *11*(4), e0153989. | 1 |
| Dlay, J. K., Duncan, G. W., Khoo, T. K., Williams-Gray, C. H., Breen, D. P., Barker, R. A., ... & Yarnall, A. J. (2020). Progression of neuropsychiatric symptoms over time in an incident Parkinson’s disease cohort (ICICLE-PD). *Brain sciences*, *10*(2), 78. | 6 |
| Elwan, M. E., Mohamed, N. R., El Shaikh, W. M., ELShereef, A. M., & Soltan, M. R. Neuropsychiatric Disorders and Sleep Disturbances in Patients with Parkinson's Disease. | 6 |
| Fox, S. H., Visanji, N., Reyes, G., Huot, P., Gomez-Ramirez, J., Johnston, T., & Brotchie, J. M. (2010). Neuropsychiatric behaviors in the MPTP marmoset model of Parkinson’s disease. *Canadian journal of neurological sciences*, *37*(1), 86-95. | 5 |
| Hatano, T., Hattori, N., Kawanabe, T., Terayama, Y., Suzuki, N., Iwasaki, Y., & Fujioka, T. (2014). An exploratory study of the efficacy and safety of yokukansan for neuropsychiatric symptoms in patients with Parkinson’s disease. *Journal of Neural Transmission*, *121*(3), 275-281. | 3 |
| Herrmann, N., Marras, C., Fischer, H. D., Wang, X., Anderson, G. M., & Rochon, P. A. (2013). Management of Neuropsychiatric Symptoms in Long-Term Care Residents with Parkinson’s Disease. *Drugs & aging*, *30*(1), 19-22. | 3,6 |
| Hidding, U., Gulberti, A., Horn, A., Buhmann, C., Hamel, W., Koeppen, J. A., ... & Pötter-Nerger, M. (2017). Impact of combined subthalamic nucleus and substantia Nigra stimulation on neuropsychiatric symptoms in Parkinson’s disease patients. *Parkinson’s Disease*, *2017*. | 5 |
| Huang, H. Y., Hsu, Y. T., Wu, Y. C., Chiou, S. M., Kao, C. H., Tsai, M. C., & Tsai, C. H. (2012). Zolpidem improves neuropsychiatric symptoms and motor dysfunction in a patient with Parkinson’s disease after deep brain stimulation. *Acta Neurol Taiwan*, *21*(2), 84-86. | 1 |
| Isais-Millán, S., Piña-Fuentes, D., Guzmán-Astorga, C., Cervantes-Arriaga, A., & Rodríguez-Violante, M. (2016). Prevalence of neuropsychiatric disorders in drug-naive subjects with Parkinson’s disease (PD). *Gac Med Mex*, *152*(3), 357-363. | 6 |
| Joyce, J. M., Monchi, O., Ismail, Z., Kibreab, M., Cheetham, J., Kathol, I., ... & Debert, C. T. (2020). The impact of traumatic brain injury on cognitive and neuropsychiatric symptoms of Parkinson’s disease. *International Review of Psychiatry*, *32*(1), 46-60. | 3 |
| Khoo, T. K., & Ioannides, Z. A. neuropsYcHiaTric sYmpToms in parkinson’s disease: WHaT rocks THe cradle? | 1 |
| Lawal, H. O., Terrell, A., Lam, H. A., Djapri, C., Jang, J., Hadi, R., ... & Krantz, D. E. (2014). Drosophila modifier screens to identify novel neuropsychiatric drugs including aminergic agents for the possible treatment of Parkinson’s disease and depression. *Molecular psychiatry*, *19*(2), 235-242. | 2 |
| Lee, W. J., Tsai, C. F., Gauthier, S., Wang, S. J., & Fuh, J. L. (2012). The association between cognitive impairment and neuropsychiatric symptoms in patients with Parkinson's disease dementia. *International psychogeriatrics*, *24*(12), 1980-1987. | 7 |
| Leiknes, I., Tysnes, O. B., Aarsland, D., & Larsen, J. P. (2010). Caregiver distress associated with neuropsychiatric problems in patients with early Parkinson’s disease: the Norwegian ParkWest study. *Acta Neurologica Scandinavica*, *122*(6), 418-424. | 6 |
| Liu, F. C., Lin, H. T., Kuo, C. F., Hsieh, M. Y., See, L. C., & Yu, H. P. (2018). Familial aggregation of Parkinson’s disease and coaggregation with neuropsychiatric diseases: a population-based cohort study. *Clinical epidemiology*, *10*, 631. | 1 |
| Löhle, M., Hermann, W., Hausbrand, D., Wolz, M., Mende, J., Beuthien-Baumann, B., ... & Storch, A. (2019). Putaminal dopamine turnover in de novo Parkinson’s disease predicts later neuropsychiatric fluctuations but not other major health outcomes. *Journal of Parkinson's disease*, *9*(4), 693-704. | 3 |
| Loiodice, S., Young, H. W., Rion, B., Méot, B., Montagne, P., Denibaud, A. S., ... & La Rochelle, C. D. (2019). Implication of nigral dopaminergic lesion and repeated L-dopa exposure in neuropsychiatric symptoms of Parkinson’s disease. *Behavioural brain research*, *360*, 120-127. | 2 |
| Mahdavi, R., Malakouti, S. K., Shahidi, G. A., & Parvaresh-Rizi, M. (2013). The Effects of Bilateral Subthalamic Nucleus Stimulation on Cognitive and Neuropsychiatric Functions in Parkinson’s Disease: A Case-Control Study. *Basic and clinical neuroscience*, *4*(3), 217. | 1 |
| Martinez-Martin P, Frades-Payo B, Agüera-Ortiz L, Ayuga-Martinez A. (2012). A short scale for evaluation of neuropsychiatric disorders in Parkinson’s disease: first psychometric approach. *Journal of neurology*, *259*(11), 2299-2308. | 7 |
| McLaughlin, N. C., Piryatinsky, I., Epstein-Lubow, G., Marino, L., & Friedman, J. H. (2014). Neuropsychiatric symptoms in an inpatient Parkinson’s disease sample. *Parkinson’s disease*, *2014*. | 6 |
| Monastero, R., Di Fiore, P., Ventimiglia, G. D., Camarda, R., & Camarda, C. (2013). The neuropsychiatric profile of Parkinson’s disease subjects with and without mild cognitive impairment. *Journal of neural transmission*, *120*(4), 607-611. | 6 |
| Mosley, P. E., & Marsh, R. (2015). The psychiatric and neuropsychiatric symptoms after subthalamic stimulation for Parkinson’s disease. *The Journal of neuropsychiatry and clinical neurosciences*, *27*(1), 19-26. | 1 |
| Mosley, P. E., Robinson, K., Coyne, T., Silburn, P., Breakspear, M., & Carter, A. (2019). ‘Woe betides anybody who tries to turn me down.’A qualitative analysis of neuropsychiatric symptoms following subthalamic deep brain stimulation for Parkinson’s disease. *Neuroethics*, 1-17. | 5 |
| Mosley, P. E., Smith, D., Coyne, T., Silburn, P., Breakspear, M., & Perry, A. (2018). The site of stimulation moderates neuropsychiatric symptoms after subthalamic deep brain stimulation for Parkinson's disease. *NeuroImage: Clinical*, *18*, 996-1006. | 3 |
| Mueller, C., Rajkumar, A. P., Wan, Y. M., Velayudhan, L., Chaudhuri, K. R., & Aarsland, D. (2018). Assessment and management of neuropsychiatric symptoms in Parkinson’s disease. *CNS drugs*, *32*(7), 621-635. | 1 |
| Munhoz, R. P., Teive, H. A., Eleftherohorinou, H., Coin, L. J., Lees, A. J., & Silveira-Moriyama, L. (2013). Demographic and motor features associated with the occurrence of neuropsychiatric and sleep complications of Parkinson's disease. *Journal of Neurology, Neurosurgery & Psychiatry*, *84*(8), 883-887. | 6 |
| Pellicano, C., Assogna, F., Cravello, L., Langella, R., Caltagirone, C., Spalletta, G., & Pontieri, F. E. (2015). Neuropsychiatric and cognitive symptoms and body side of onset of parkinsonism in unmedicated Parkinson's disease patients. *Parkinsonism & related disorders*, *21*(9), 1096-1100. | 6 |
| Peralta, C. (2012). Neuropsychiatric non motor symptoms of Parkinson's disease. *Vertex (Buenos Aires, Argentina)*, *23*(106), 428-432. | 4 |
| Rektorová, I. (2010). Effects of dopamine agonists on neuropsychiatric symptoms of Parkinson’s disease. *Neurodegenerative Diseases*, *7*(1-3), 206-209. | 6 |
| Rodríguez-Constenla, I., Cabo-López, I., Bellas-Lamas, P., & Cebrián, E. (2010). Trastornos cognitivos y neuropsiquiátricos en la enfermedad de Parkinson. *Rev Neurol*, *50*(Supl 2), S339. | 1 |
| Schmitt, E., Krack, P., Castrioto, A., Klinger, H., Bichon, A., Lhommée, E., ... & Martinez‐Martin, P. (2018). The neuropsychiatric fluctuations scale for Parkinson's disease: a pilot study. *Movement disorders clinical practice*, *5*(3), 265-272. | 5 |
| Scorza, F. A., Gadelha, A., Ferraz, H. B., Finsterer, J., & Bressan, R. A. (2017). Beyond the neuropsychiatric horizon: assessing the risk of sudden unexpected death in Parkinson disease. *Journal of the American Medical Directors Association*, *18*(11), 988. | 1 |
| Taylor, J., Anderson, W. S., Brandt, J., Mari, Z., & Pontone, G. M. (2016). Neuropsychiatric complications of Parkinson disease treatments: importance of multidisciplinary care. *The American Journal of Geriatric Psychiatry*, *24*(12), 1171-1180. | 1 |
| Thippeswamy, H., Viswanath, B., Babu, G. N., Reddi, V. S. K., & Chaturvedi, S. K. (2014). Consultation-liaison approach for the management of psychiatric manifestations in Parkinson's disease and related disorders: A report from Neuropsychiatric Hospital, India. *Indian journal of psychological medicine*, *36*(2), 134-137. | 6 |
| Tsai, C. H., Huang, H. C., Liu, B. L., Li, C. I., Lu, M. K., Chen, X., ... & Lane, H. Y. (2014). Activation of N‐methyl‐D‐aspartate receptor glycine site temporally ameliorates neuropsychiatric symptoms of P arkinson's disease with dementia. *Psychiatry and clinical neurosciences*, *68*(9), 692-700. | 7,5 |
| Tsai, W. C., Lin, H. C., Chang, C. C., Chang, W. N., Huang, C. C., Cheng, K. Y., ... & Tsai, N. W. (2020). Neuropsychiatric symptoms in Parkinson’s disease: association with caregiver distress and disease severity. *International psychogeriatrics*, *32*(6), 733-739. | 5,6 |
| van Balkom, T. D., Vriend, C., Berendse, H. W., Foncke, E. M., van der Werf, Y. D., van den Heuvel, O. A., & Klein, M. (2016). Profiling cognitive and neuropsychiatric heterogeneity in Parkinson’s disease. *Parkinsonism & related disorders*, *28*, 130-136. | 3 |
| Wang, H. T., Wang, L., He, Y., & Yu, G. (2018). Rotigotine transdermal patch for the treatment of neuropsychiatric symptoms in Parkinson's disease: A meta-analysis of randomized placebo-controlled trials. *Journal of the neurological sciences*, *393*, 31-38. | 1 |
| Weintraub, D., Caspell‐Garcia, C., Simuni, T., Cho, H. R., Coffey, C. S., Aarsland, D., ... & Parkinson’s Progression Markers Initiative. (2020). Neuropsychiatric symptoms and cognitive abilities over the initial quinquennium of Parkinson disease. *Annals of clinical and translational neurology*, *7*(4), 449-461. | 6 |
| Weintraub, D., Simuni, T., Caspell‐Garcia, C., Coffey, C., Lasch, S., Siderowf, A., ... & Parkinson's Progression Markers Initiative. (2015). Cognitive performance and neuropsychiatric symptoms in early, untreated Parkinson's disease. *Movement Disorders*, *30*(7), 919-927. | 6 |
| Willis, A. W., Schootman, M., Kung, N., & Racette, B. A. (2013). Epidemiology and neuropsychiatric manifestations of Young Onset Parkinson's Disease in the United States. *Parkinsonism & related disorders*, *19*(2), 202-206. | 6 |
| Witt, K., Daniels, C., & Volkmann, J. (2012). Factors associated with neuropsychiatric side effects after STN-DBS in Parkinson's disease. *Parkinsonism & related disorders*, *18*, S168-S170. | 1 |
| Yao, M. L., Zhang, H., Xu, Y., Zhang, S. M., Gao, Y. Z., Shu, M., & Zhang, J. J. (2019). Neuropsychiatric Symptoms and Cognitive Impairment in Chinese Patients with Parkinson’s Disease in Han and Hui Ethnicity. *Current medical science*, *39*(1), 122-126. | 6 |
| Ye, B. S., Jeon, S., Yoon, S., Kang, S. W., Baik, K., Lee, Y., ... & Sohn, Y. H. (2018). Effects of dopaminergic depletion and brain atrophy on neuropsychiatric symptoms in de novo Parkinson’s disease. *Journal of Neurology, Neurosurgery & Psychiatry*, *89*(2), 197-204. | 6 |

^†^Exclusion criteria: 1. Reviews or non-experimental articles; 2. Animal studies; 3. Reports not directly related to the research objectives; 4. Reports not available through the specified databases; 5. Participants sample <30; 6. Reports of patients with PD illness duration after clinical diagnosis <3 years; 7. Reports of patients diagnosed with any other disorder that could interfere in the final results.
